# Occurrence and Dynamism of Lactic Acid Bacteria in Distinct Ecological Niches: A Multifaceted Functional Health Perspective

**DOI:** 10.3389/fmicb.2018.02899

**Published:** 2018-11-27

**Authors:** Fanny George, Catherine Daniel, Muriel Thomas, Elisabeth Singer, Axel Guilbaud, Frédéric J. Tessier, Anne-Marie Revol-Junelles, Frédéric Borges, Benoît Foligné

**Affiliations:** ^1^Université de Lille, Inserm, CHU Lille, U995 – LIRIC – Lille Inflammation Research International Center, Lille, France; ^2^Université de Lille, CNRS, Inserm, CHU Lille, Institut Pasteur de Lille, U1019 – UMR 8204 – CIIL – Center for Infection and Immunity of Lille, Lille, France; ^3^Micalis Institute, INRA, AgroParisTech, Université Paris-Saclay, Jouy-en-Josas, France; ^4^Laboratoire d’Ingénierie des Biomolécules, École Nationale Supérieure d’Agronomie et des Industries Alimentaires – Université de Lorraine, Vandœuvre-lès-Nancy, France

**Keywords:** lactic acid bacteria, gut microbiota, fermented food, probiotics, ecosystems, ecological niches

## Abstract

Lactic acid bacteria (LAB) are representative members of multiple ecosystems on earth, displaying dynamic interactions within animal and plant kingdoms in respect with other microbes. This highly heterogeneous phylogenetic group has coevolved with plants, invertebrates, and vertebrates, establishing either mutualism, symbiosis, commensalism, or even parasitism-like behavior with their hosts. Depending on their location and environment conditions, LAB can be dominant or sometimes in minority within ecosystems. Whatever their origins and relative abundance in specific anatomic sites, LAB exhibit multifaceted ecological and functional properties. While some resident LAB permanently inhabit distinct animal mucosal cavities, others are provided by food and may transiently occupy the gastrointestinal tract. It is admitted that the overall gut microbiome has a deep impact on health and diseases. Here, we examined the presence and the physiological role of LAB in the healthy human and several animal microbiome. Moreover, we also highlighted some dysbiotic states and related consequences for health, considering both the resident and the so-called “transionts” microorganisms. Whether LAB-related health effects act collectively or follow a strain-specificity dogma is also addressed. Besides the highly suggested contribution of LAB to interplay with immune, metabolic, and even brain-axis regulation, the possible involvement of LAB in xenobiotic detoxification processes and metal equilibrium is also tackled. Recent technological developments such as functional metagenomics, metabolomics, high-content screening and design *in vitro* and *in vivo* experimental models now open new horizons for LAB as markers applied for disease diagnosis, susceptibility, and follow-up. Moreover, identification of general and more specific molecular mechanisms based on antioxidant, antimicrobial, anti-inflammatory, and detoxifying properties of LAB currently extends their selection and promising use, either as probiotics, in traditional and functional foods, for dedicated treatments and mostly for maintenance of normobiosis and homeostasis.

## Introduction

A historical metabolic-based (and somewhat pleonasmic) consensus definition of lactic acid bacteria (LAB) is a broad group of bacteria characterized by the formation of lactic acid as a sole or main (over 50%) end product of carbohydrate utilization. However, LAB more strictly correspond to members of the order *Lactobacillales* from a taxonomic point of view. The important taxonomic and physiological diversity of LAB representatives is, however, not convenient when addressing specific ecological niches and roles and applications of LAB. Indeed, LAB adapt to various conditions and change their metabolism accordingly; they cover a varied range of genera including species of lactobacilli, enterococci, lactococci, pediococci, streptococci, tetragenococci, vagococci, leuconostocs, oenococci, carnobacteria, and weissella. The LAB thus constitutes a very heterogeneous group and are often misleadingly circumscribed to lactobacilli only. In contrast, other microbes used in the making of fermented dairy products or claimed as probiotics such as bifidobacteria, propionibacteria, and even brevibacteria, belonging to the anaerobic actinomycetales, are falsely included or assimilated within this group. This is partly due to their overlapping habitat or common properties together with unclear species identity.

Recent efforts have been undertaken to identify lactobacilli and related species (Sun et al., 2015). From eighty identified *Lactobacillus* species, 15 years ago (Satokari et al., 2003), we now reach to more than 200, with continuous new discoveries, e.g., *Lactobacillus timonensis* (Afouda et al., 2017) or *Lactobacillus metriopterae* (Chiba et al., 2018) due to next-generation sequencing (NGS), clustered regularly interspaced short palindromic repeats (CRISPR)-based methods (Sun et al., 2015), and culturomics (Lagier et al., 2015). This has justified the proposal for a reclassification of the genus (Claesson et al., 2008). According to the last taxonomic update of the lactobacilli, a dozen clades have been recently organized (Salvetti et al., 2012). The current knowledge on evolution of the genus *Lactobacillus*, its environmental niches, and the degree of host specificity was recently completed (Duar et al., 2017). Ecological differentiation of the genus *Carnobacterium* was recently established based on comparative genomic analysis (Iskandar et al., 2017).

The genus *Enterococcus* encompasses more than 50 species that can also be found in diverse environments, from the soil to the intestine of animals and humans, including the hospital environment, which provide concerns (Dubin and Pamer, 2014). In addition, enterococci are present as spoilage microflora of processed meats, but, on the other hand, they are important for aroma development and ripening of traditional products such as certain cheeses and sausages (Franz et al., 2003). Depending on their origins and evolution (Lebreton et al., 2013), they may act as both commensals and pathogens, and strains are consequently of clinical importance, harboring virulence factors (Ali et al., 2017) as well as possibly being used as probiotics (Holzapfel et al., 2018).

The various genera, species and even strains of LAB inhabit and cope with specific environments in order to exert dedicated or multiple specific functions according to their structural determinants and/or metabolic pathways. With the exception of enterococci as opportunistic pathogens and the case of pathogenic streptococci, there are only a few reported cases of bacteremia due to LAB (Goldstein et al., 2015; Kamboj et al., 2015). The LAB are regarded as generally recognized as safe (GRAS) because of their ubiquitous use in food and their unique role in the healthy microflora of human mucosal surfaces. To date, 50 LAB strains have obtained the qualified presumption of safety (QPS) status by the European Food Safety Agency (Ricci et al., 2017), again, comprising mostly lactobacilli *spp.* (*n* = 37). However, enterococci have been classified in risk group 2 in the European Directive 93/88.

The LAB greatly differ in morphology, optimal growth and tolerance temperature, salt and pH tolerance, metabolism, and surface and secreted molecule (Lebeer et al., 2010; Pessione, 2012; Gänzle, 2015). They may secrete effector proteins, produce exopolysaccharides (EPS) and generate biofilms, or adhere on abiotic and/or biologic surfaces, depending upon genera, species, or strain. Accordingly, the origins, quantities, the diversity, and abundances of LAB strains in complex ecosystems may greatly impact on the intended effect(s), product(s), and functionality. As a striking example of broad effects, the presence of psychrotrophic *Lactobacillus* spp. as the prevailing spoilage organisms in packaged cold-stored meat or fish products is unwanted (Andreevskaya et al., 2018). In contrast, dominance of only a few *Lactobacillus* species inhabiting human vaginal cavities is essential to maintain a low microbial diversity and prevent further vaginosis (Borges et al., 2014; Vaneechoutte, 2017b). Consequently, LAB, in general, and lactobacilli, in particular, cannot be considered as a whole, and have to be stratified depending on their intrinsic characteristics and potential applications. Hence, up to 25 species are defined as fructophilic LAB that inhabit fructose-rich niches in nature, e.g., flowers, fruits, and fermented foods. These LAB can also be isolated from the gut of bees and flies (Endo, 2012), and the latter have coevolved to determine microbe–host mutualism (Matos and Leulier, 2014). A motile phenotype has been characterized in nearly 15 distinct *Lactobacillus* species, while motility genes have also been detected in other closely related strains. This will both contribute to select geographical ecological niches and, due to flagellin signalization, to sustain the immune potential of such bacteria (Cousin et al., 2015).

It has been recognized for a long time that LAB are fascinating and useful bacteria (Tannock, 2004) and, now, recent advances in technologies allow addressing the specific mechanisms of the important bacteria–host interactions in appropriate models. The uses of LAB as biotherapeutic agents and probiotics are based on multiple molecules and modes of action that are currently deciphered. Here, we present a short overview dealing with the overall occurrence of LAB in the environment and some of their contributions to health, focusing on strain-dependent effects together with consideration on individual hosts and experimental models.

## Lab in the Environment and Raw and Fermented Foods

The LAB can be found nearly everywhere although their total load and relative abundance in microbial ecosystems are extremely diverse and depend on the specific environment. The selective pressure exerted by these environments is a key driver in the genomic diversity among LAB strains derived from distinct habitats (McAuliffe, 2018). LAB have been identified in Japanese lakes, with viable cell counts ranging from 1 to 3 log per ml, with a clear seasonal variation (Yanagida et al., 2007). LAB could also be isolated from soils following enrichment protocols (Chen et al., 2005). Although soils do not contain large amounts of LAB, they are somewhat more abundant on the plant–soil interface such as the rhizosphere of some trees and comestible fungi. Some of the strains obtained from these niches showed antimicrobial properties (Fhoula et al., 2013). The abundance and diversity of LAB in soils greatly depend on carbon-richness, which is, e.g., greater under fruit–trees and in soils associated with anthropic activities or free-range farming, and after the use of manure. Notably, many halo-tolerant LAB, known to be able to survive and even grow in dry environments, can be recovered from soils when soil acidity may contribute too. It is interesting to note that LAB are also used in agriculture, as biofertilizers, safe biocontrol agents for bacterial and fungal phytopathogens, regulators of abiotic plant stress, and biostimulant agents to ameliorate plant growth (recently reviewed by Lamont et al., 2017). Understanding the phytomicrobiome, including the role of LAB therein is an emerging field with great potential to mitigate plant stress and promote plant resistance and production.

The LAB only represent a subdominant part of raw vegetables and fruits microorganisms (2 and 4 log cfu per g.), while the microbial autochtonous population varies between 5 and 7 log cfu per g. (Di Cagno et al., 2013); hetero-fermentative and homo-fermentative species belonging to *Leuconostoc*, (mostly *L. plantarum*), *Weissella*, *Enterococcus*, and *Pediococcus* genera are those most frequently identified as epiphytes within the microbiota, depending on the plant species. We also should consider the low but relevant endophyte LAB representatives when regarding adaptation of LAB to plant niches. Indeed, the capacity to adapt to the intrinsic features of the raw plant matrices and persist stably as endophytes throughout plant phenological stages represent additional criteria for selecting robust LAB candidates (Filannino et al., 2018). Some LAB found in bakery sourdough are there partly due to contaminating flour and may originate from milling, representing a part of the endophytic microbial community of wheat at very low levels. Moreover, LAB diversity in sourdoughs may also originate from external layers of wheat plant organs (epiphytic) and the bakery environment (the bakers, insects, and nuisance species animals) (Minervini et al., 2015). The ability of LAB to produce organic acids and other antimicrobial substances has made them essential in the preservation of plant-based foods while they also are the most important microbes promoting significant positive changes in healthy aspects of plant foods. Their metabolism throughout fermentation contributes to lowering some toxic and antinutritional factors and promoting bioavailable bioactive compounds (Di Cagno et al., 2013; Marco et al., 2017; Filannino et al., 2018). Following either spontaneous fermentation or after intentional inoculation of food products with LAB as starters, the final LAB load in plant products can reach 8 to 9 log per g. Such a high concentration of LAB contributes to the biocontrol of pathogens (Gram-negative bacteria, *Listeria monocytogenes*) in food as well as to their possible use in probiotic/functional food.

The natural origin of LAB used in traditional fermented foods, such as sauerkraut, pickles, cheese, sausage, fish, fish- and soy- sauce, sourdough bread, and animal silage, is the corresponding matrix (fruits, cereals, milk, and animal farm environment) or the associated (wooden) material (Lortal et al., 2014). A plant-based origin for dairy lactococci was nicely demonstrated using genome evolution studies (McAuliffe, 2018). Starters and ferments have then been isolated, selected, and domesticated over past centuries to control the fermentation processes and standardize the taste and quality of the final products. The final count of LAB in fermented products is highly diverse and ranges from 4 to 5 log to over 9 log of bacteria, depending on product types, fermentation dynamics, and the overall microbial ecology of the products. Moreover, the abundance of other non-LAB, yeasts, and molds in these complex ecosystems, especially in cheeses, needs to be considered. The microbial diversity of fermented food together with some functional properties, including the contribution of some LAB therein, has been extensively reviewed elsewhere (Tamang et al., 2016a,b; Linares et al., 2017; Marco et al., 2017). The health benefits of fermented foods and the added value of ingesting LAB in order to either prevent or treat some specific diseases is now generally accepted and beyond discussion. Regardless of their traditional or industrial origins, fermented foods are active sources of LAB, among other microbes, that will enter the digestive tract and possibly exert a positive influence (and even a negative one) on the host.

## Occurrence of Lab in Mucosal Niches

### The Human Vaginal Tract as a Unique Example of LAB Dominance and Functionality

Dominance of LAB in the vaginal niche is a characteristic of healthy women, reaching from 70 to up to 90% of resident bacteria, whereas LAB colonization of the genital tract of other mammal species, including primates, is anecdotic and generally below 1% of the relative abundance (Miller et al., 2016). This is unique in humans where vaginal fluid generally contains 7 to 8 log of lactobacilli per mL, represented by a dozen or so species most frequently found (Borges et al., 2014). However, the normal vaginal flora is usually dominated by one or two out of the major species of lactobacilli (Vaneechoutte, 2017b). In addition, occurrence of other rare *Lactobacillus* species and other LAB representatives such as *Pediococcus acidilactici*, *Weissella* spp. (*W. kimchi, W. viridescens*), *Streptococcus anginosus*, and *Leuconostoc mesenteroides* has also been reported. These observations greatly depend on ethnic groups, individuals, and time, according to pregnancy or menopausal status (Borges et al., 2014). Notably, not all *Lactobacillus*-deficient vaginal microbiotas are adverse (Doyle et al., 2018). The main benefits of lactobacilli in the vaginal sphere are essentially that they have anti-infectious properties, and can prevent and target bacterial vaginosis, vaginal candidiasis, and sexually transmitted virus and bacterial pathogens. The normobiosis of the vaginal microbial community is based on low pH maintenance (ranging from 3.5 to 4) due to lactate production following glycogen consumption, which is unique in humans, and not necessarily in other animals. Another key factor is hydrogen peroxide production. While 95% of lactobacilli of vaginal origin from healthy women are H_2_O_2_-producers, this proportion can drop to 6–20% in the context of vaginosis. Moreover, dominance of *L. iners*, a non-H_2_O_2_ producer, has been associated with poor protection and even a risk-factor in vaginal dysbiosis (Vaneechoutte, 2017a). However, the specific role of *L. iners*, which is uncultivable in standard LAB culture media and unable to produce D-lactate, is still on debate (Petrova et al., 2017). Finally, bacteriocin production is also involved in direct killing of microorganisms by lactobacilli in the vagina although most vaginal LAB isolates do not exhibit bactericidal activity (Spurbeck and Arvidson, 2011). Anyway, this production is an optional issue in selecting appropriate strains and sustaining the role of probiotics in maintaining vaginal health (Borges et al., 2014). As a more indirect mechanism, a recent study has demonstrated that the overall *Lactobacillus*-associated anti-inflammatory properties in the vaginal mucosa could contribute to lower HIV infectiveness (Gosmann et al., 2017). Collectively, only few LAB species are adapted to dominate the human vaginal cavity in order to preserve health.

### Heterogeneity of LAB Abundance Within the Gut Microbiota

Very recently, Heeney et al. (2018) raised the question of the importance of *Lactobacillu*s in the intestine of mammals by reviewing the occurrence of LAB in the gastrointestinal tract of distinct vertebrates. In contrast to rodents where LAB can represent 20% to up to 60% of all bacteria, the estimated proportion of LAB in the human proximal small intestine is always subdominant (6%), while their relative abundance in the colon is mostly below 0.5% or non-detectable. LAB have also been detected in substantial amounts in the stomach of humans although the actual numbers are subject to huge variations depending on individuals and their health status, the methodology used, and the type of samples. Depending on genetic backgrounds, the gut microbiota of laboratory mice is highly diverse and the proportion of LAB can range from undetectable to near 100%, ranging from 4.7 to 10.6 log (Friswell et al., 2010; Nguyen et al., 2015). The gut microbiome of wild wood mice comprises an average of 30% of *Lactobacillales*, although it dramatically changes with the seasons. Indeed, the proportion of *Lactobacillus* spp. ranges from 60% in spring to 20% in the fall (Maurice et al., 2015). The abundance of LAB in the intestinal microbiota of laboratory mice is highly influenced by the diet and can range from 10% *Lactobacillales* on a normal fiber control diet to 0.5% with low-fiber diet and 40% with a high-fiber diet (Trompette et al., 2014). Similarly, LAB abundances also vary according to circadian rhythm disturbances (Voigt et al., 2014).

The mode of delivery (vaginal versus C-section) in humans is important to allow, respectively, high or low levels of *Lactobacillales* in babies. These differences persist during the first 2 years of life and progressively disappear (Dominguez-Bello et al., 2010). Indeed, lactobacilli and enterococci are first colonizers and dominant bacteria, then become subdominant at toddler and adult ages. A metagenomic study identified nearly 60 distinct species of lactobacilli in human fecal samples, not exceeding 0.04% of all bacteria present, corresponding to near 8.5 log cell per g. from an average bacterial load of 12 log (Rossi et al., 2016). Within this diversity, one or two major species (*L. rhamnosus* and *L. acidophilus)* were estimated at 8 log. Of the six most represented species, those estimated from 7.5 log were identified as *L. rhamnosus, L. ruminis, L. acidophilus, L. delbrueckii, L. casei*, and *L. plantarum*, while others were detected below 5 log cell per g., close to the detection threshold. In this study, the abundance of enterococci was similar to that of lactobacilli, but commensal streptococci were two to five times more abundant. Turroni et al. (2014) reported dominance of *L. gasseri, L. casei, L. namurensis*, *L. rogosae*, and *L. murinus* in human fecal samples. Clearly, proportions of LAB and even *Lactobacillus* species are highly diverse and vary between individuals (Booijink et al., 2010). This may explain difficulties at comparing microbiome data from healthy controls and patients with distinct pathologies aiming at uncovering a possible protective or even deleterious general role of lactobacilli. Many studies investigating the role of the human gut microbiota in distinct immune-mediated inflammatory diseases have reported either increased, decreased, or unchanged levels of lactobacilli (Forbes et al., 2016; Heeney et al., 2018). All in all, no consistent marker for any pathology or a healthy state is simply defined by a specific proportion of *Lactobacillus*, or even based on the follow-up of the *Lactobacillus* load for an individual in time. Interestingly, gut remodeling due to a restrictive surgery and leading to short bowel syndrome was accompanied by enrichment of lactobacilli (Joly et al., 2010; Boccia et al., 2017), which may explain the D-lactate acidosis in some subgroups of patients (Joly et al., 2010; Mayeur et al., 2016; Boccia et al., 2017). The in lactobacilli-enriched microbiota in patients suffering from short bowel syndrome may also favor energy recovery occurring after resection (Gillard et al., 2017). The abundance of enterococci in the human gut, mostly *E. faecium* and *E. faecalis*, is estimated between 4 and 6 log bacteria per g. wet weight (Layton et al., 2010). It, thus, represents nearly 1% of the relative abundance, which is 10–100 times higher than that of lactobacilli. Although commensal LAB are somewhat subdominant, they clearly play a role in gut physiology with consequences on health and qualitative (species and strain specificity) rather than quantitative aspects (genera and family abundance) should be considered. The interplay between LAB and non-LAB within the gut has not been completely revealed yet. For instance, several authors have highlighted the possible indirect role of LAB in increasing the butyrate content in the feces. This capability is attributed to an initial cleavage of fibers in the large intestine to more fermentable compounds, which are then further converted to butyrate by butyrogenic non-LAB present. Moreover, exogenous LAB, sourced after oral administration of fermented foods or dietary supplements, may reach numbers similar to those of commensals (0.1–1% of the total microbiota present in the gastrointestinal tract, so 5–8 log per g.), depending on dietary habits and geographic areas (Plé et al., 2015). Such transitory or “visiting” living microorganisms, also named pseudocommensals (Rook, 2013) or transionts, cannot durably colonize the host. However, provided these bacteria are regularly or even occasionally ingested in sufficient amounts, they can act as symbionts or pathobionts, modulate the gut microbiota, and exert various health effects.

### The Lung as an Emerging Example of LAB Occurrence and Functionality?

The exploration of the microbiota in the lung is in its infancy when compared with the microbiomes from the gut or the vaginal cavity. Using 16S metagenomics, the microbiota of the respiratory tract has been described since 2010; it is mainly made up by two phila (Firmicutes and Bacteroidetes) in humans and four phila (Proteobacteria, Firmicutes, Bacteroidetes, and Actinobacteria) in mice (Singh et al., 2017). The load of lung microbiota is estimated to reach around 10^3^–10^4^ cultivable bacteria per gram of lung homogenates in mice (Remot et al., 2017). In humans, it has been estimated as a mean of 3 log bacterial genomes per cm^2^ surface in the upper lobe (Hilty et al., 2010). The lung microbiota plays a key role in promoting tolerance (Gollwitzer et al., 2014) and is a determinant of maintenance of homeostasis. It remains difficult to define a healthy lung microbiota as of yet, due to its high intervariability as a function of age, diet, or environment, but LAB (*Streptococcus*, *Lactobacillus*, and *Enterococcus*) are prominent members and seem to be actors of respiratory symbiosis and health. As an example, the presence of *Enterococcus faecalis* decreases with the severity of asthma in the human lung microbiota (Turturice et al., 2017) and the decrease of the bacteria improves the outcomes of asthma in a preclinical mouse model (Remot et al., 2017). Nasal administration of *Lactobacillus rhamnosus* GG protects against influenza virus infection (Harata et al., 2010) and oral supplementation of *Lactobacillus* spp also modifies the lung ecosystem. Overall, these data indicate that LAB are major members of lung microbiota and might be useful in future preventive strategies or new therapeutics for respiratory health.

## Regardless of Their Origin, Lab Can Exhibit Multifaceted Functional Properties

### Immune Properties

Anti-inflammatory properties of LAB have been extensively studied in rodents and to a lesser extent in humans (Hevia et al., 2015; Papadimitriou et al., 2015). This is attested by many studies sustaining the protective role of commensal LAB and the benefits brought by exogenous LAB through nutritional and/or probiotic interventions. The overall anti-inflammatory value of LAB is also evidenced by the rarefaction of lactobacilli in the gut of inflammatory bowel disease patients. Moreover, the drop of lactobacilli observed in the fecal microbiota of aging populations can be related to the low-grade inflammation theory, i.e., ‘inflamm-aging’ and frailty (van Tongeren et al., 2005; Franceschi et al., 2017). Recently, it was shown that diet-induced exacerbation of experimental colitis is associated with a reduction in *Lactobacillus* sp. and a lower production of protective short-chain fatty acids, including butyrate (Miranda et al., 2018). Nevertheless, whether the overall rate of LAB or even a minimal threshold of lactobacilli may or may not represent an indicator for health is not yet clear. The prevalence and richness of *Lactobacillales* and lactobacilli may either increase or decrease depending on immune disease types and studies (Forbes et al., 2016; Heeney et al., 2018), and such changes can be linked to causal events or adaptive processes to counteract the injury. Again, besides genus and species, also strain-level attributes matter when considering immunomodulatory properties of LAB (Sanders, 2007). This has been well documented during the last decades, showing anti-inflammatory probiotic properties of specific lactobacilli based on multiple distinct mechanisms of reducing colitis symptoms in mice (Sanders et al., 2018). For example (non-exhaustive), a *L. plantarum* strain was shown to be beneficial against inflammation because of a specific teichoic acid structure (Grangette et al., 2005), while the anti-inflammatory effect of a strain of *L. salivarius* was dependent on peptidoglycan (Macho Fernandez et al., 2011). In contrast, the alleviation of colitis was attributed to S-layer proteins of *L. acidophilus* (Konstantinov et al., 2008), pili for a *L. rhamnosus* strain (Lebeer et al., 2012), and EPS for another *L. plantarum* (Górska et al., 2014). In line with these observations, the structurally different EPS from resident lactobacilli generate different immune responses by dendritic cells while, upon gut inflammation, specific bacterial molecular motifs are absent from lactobacilli isolated from IBD (Górska et al., 2016), providing tools for further application based on strain selection (Oleksy and Klewicka, 2018). The positive role of H_2_O_2_ production to lower inflammation has been reported for *L. crispatus* and *L. rhamnosus* (Voltan et al., 2008; Lin et al., 2009). The colitis alleviating property of *L. bulgaricus* was related to activation of the aryl hydrocarbon receptor pathway in colon cells (Takamura et al., 2011), in line, the control of inflammation by an *L. reuteri* intervention was associated to the production of histamine followed by activation of a host epithelial cell receptor (Gao et al., 2015). An *L. casei* strain inhibited the secretion of the pro-inflammatory mediator IP-10 protein at the post-translational level (Hoermannsperger et al., 2009); this inhibition is based on the role of a specific secreted protein by the bacteria (von Schillde et al., 2012). Specific bacterial DNA motifs may also drive some of the immune-stimulatory effects in a toll-like receptor 9-dependent manner (Iliev et al., 2008). Interestingly, distinct immunological activities through TLR5-signaling caused by flagellins isolated from motile lactobacilli presume a consequence of adaptation to commensalism (Kajikawa et al., 2016). Pro-inflammatory LAB, i.e., *L. crispatus*, were inconsistently reported in murine models of colitis (Zhou et al., 2012). Given that these observations are rare, one has to keep in mind that such undesirable (and embarrassing) results are difficult to publish and that they may be underestimated. LAB other than lactobacilli can also exert immune effects and strain-dependent impact on the release of pro-inflammatory cytokines and colitis. Strain diversity in such anti-inflammatory properties has been reported for food-derived pediococci and oenococci (Foligné et al., 2010), carnobacteria (Rahman et al., 2014), and enterococci (Wang et al., 2014). Notably, several strains of *Enterococcus spp*. are marketed as probiotics, for use in many health purposes both in humans and pets, alone or in combination with other LAB and/or bifidobacteria but most of the *Enterococcus* species are believed to have no prominent beneficial effect on inflammation as well as on the overall human health.

No single and unique mechanism of anti-inflammatory effects of LAB can be generalized. The overall combination of anti-inflammatory and anti-oxidative properties with the occurrence of some immune-stimulatory signals of a single LAB strain has to be integrated by the host. Moreover, when interpreting the host’s health, one also should consider the interplay of these specific molecular players with those of other (non-)LAB strains from the microbiota. Consequently, the anticipated effects of a promising strain can either be boosted or diluted, depending on its microbial microenvironment in the gut. In addition, the contribution of the matrix should not be neglected (Burgain et al., 2014; Lee et al., 2015). Together with a high variability in host immune reactivity, it seems difficult to fully predict the performance of individual LAB strains. It is essential to screen LAB strains with valuable properties (Foligné et al., 2013; Papadimitriou et al., 2015), but integrative endpoints are necessary to fully characterize the consequences of LAB for health; relevant anti-inflammatory strain selection should thus be based on specific mechanisms and how they may interplay within the microbiota (Lebeer et al., 2018).

### Metabolic Properties

Clear links have been established between gut microbiota, metabolism, and the nutritional status of distinct animals, including farm animals, whose growth performance was empirically boosted either by antibiotics or probiotics for several decades. The key role of gut microbes in the metabolic physiology of the host, throughout evolution, has been demonstrated in drosophila (Leulier and Royet, 2009) in fish (Egerton et al., 2018), in rodents and humans (Ley et al., 2006; Gérard, 2016). Molecular mechanisms involved multiple signaling pathways such as microbial production of short-chain fatty acids, the control of epithelial integrity (and endotoxemia), and modulation of chronic inflammation, which has been reviewed elsewhere (Maruvada et al., 2017; Dao and Clément, 2018). Microbiota-derived metabolites can also interplay with the regulation of appetite and satiety. They act particularly on intestinal food intake mediators likely GLP1, leptin, and ghrelin. In mammals, disruption of the homeostasis of gut microbiota (dysbiosis), resulting from an imbalance of bacterial strains, may induce physio-pathological processes leading to chronic obesity or metabolic disorders such as type 2 diabetes or metabolic syndromes (Carding et al., 2015).

Gnotobiotic rodents have been used to study the health effects on germ-free (axenic) animals of treatment with specific bacterial strains. The glycolytic activity of *Streptococcus thermophilus* is improved once inside the digestive tract of mono-associated rats (Rul et al., 2011). Colonization of the gut of germ-free mice by microbiota from obese mice significantly increases their total body fat compared with colonization by microbiota from lean mice (Turnbaugh et al., 2006). In addition, inoculation of both obese mice and humans with microbiota from lean mice or humans, respectively, improves symptoms of metabolic syndrome (Vrieze et al., 2012; Kulecka et al., 2016; Ji et al., 2018). Some *Lactobacillus* species are associated with weight gain, while others are associated with protection against obesity (Drissi et al., 2014). Compared to lean patients with a normal body mass index, abundance of lactobacilli was higher in obese and lower in anorexic individuals (Ley et al., 2005; Armougom et al., 2009). In contrast, a recent study established a relationship between the high oral *Lactobacillus* counts and protection to further weight gain, while a lack or a low level of oral lactobacilli may increase the risk of obesity (Rosing et al., 2017). Higher proportions of lactobacilli were related with type 2-diabetes (Larsen et al., 2010; Karlsson et al., 2013). Moreover, some lactobacilli were also reported to limit undernutrition and to have growth-promoting effects in mice (Schwarzer et al., 2016) while a strain of *L. reuteri* could contribute to preventing cachexia (Bindels et al., 2016). Data dealing with the occurrence of *Lactobacillus* spp. in obesity and type 2-diabetes are thus inconsistent, which is most probably related to their low and variable quantity in the gut microbiota. Comparative genomic analyses have shown that *Lactobacillus* species linked to weight-loss had specific arsenals of genes associated with anti-microbial activities such as bacteriocins (Drissi et al., 2014). In contrast, weight gain-associated *Lactobacillus* spp. harbored enzymes involved in lipid metabolism. Besides the species level, the importance of properties at the strain level was revealed using interventions in experimental rodent models.

Significant research efforts over the recent decades have been devoted to the development of effective treatments for obesity and metabolic disorders, using probiotics to mitigate dysbiosis and its impact on metabolism. Several studies have shown that ingestion of LAB by rodents reduced weight gain and improved the metabolic profile (blood glucose level, insulin, leptin), oxidative stress, and hepatic inflammation in various models such as mice fed with a high fat diet (HFD) (Alard et al., 2016; Park et al., 2017); Lepr*^db/db^* mice (lacking the functional, full-length Ob-Rb leptin receptor) (Yun et al., 2009), streptozotocin (STZ)-induced diabetic mice fed an HFD (Pei et al., 2014), STZ-diabetic rats (Tabuchi et al., 2003), and rats fed with a diet high in fructose (Hsieh et al., 2013). These experiments mostly underlined the role of *L. rhamnosus*, *L. plantarum, L. gasseri*, *L. casei L. mali, L. fermentum*, and *L. reuteri* strains alone or in combination with other strains. Again, not all *Lactobacilli* are able to control obesity and adiposity and some, i.e., an *L. salivarius* strain known as a probiotic for anti-inflammatory properties in mice, could not alleviate diet-induced obesity and insulin resistance, while a strain of *L. rhamnosus* did (Alard et al., 2016). Similar results have also been observed in clinical trials. For example, glycemia and cholesterol levels were reduced in elderly subjects after a month of daily consumption of a combination of *L. acidophilus* and *B. bifidum* strains (Moroti et al., 2012), while a reduction was observed of oxidative stress and glycemia levels in type-2 diabetes patients after 6 weeks of daily consumption of a probiotic dairy product containing *L. acidophilus* and *B. lactis* (Ejtahed et al., 2012). The beneficial effects of various species of *Lactobacilli* on obesity throughout clinical interventions have not been demonstrated nor reviewed yet (Crovesy et al., 2017). One possible explanation of metabolic disorders alleviation is that probiotics, and especially LAB, could reduce the absorption and conversion of food into useable energy and subsequent fat storage, resulting in anti-obesity, anti-inflammatory, and anti-diabetic effects in mice and humans (Kerry et al., 2018). The current molecular hypothesis of anti-obesity mechanisms involves the specific role of short-chain fatty acids and the host cell receptors FFAR2 and FFAR3, the contribution of bile-salt hydrolases from LAB, and further bile signature signaling of FXR and TGR5 receptors as well as LAB metabolites as antagonists of AhR (Lamas et al., 2016). However, no clear explanation is achieved yet.

In addition, LAB strains that either lower pathogen or even pathobiont numbers, those that strengthen the gut barrier and reduce LPS-endotoxemia, and the anti-inflammatory strains are also valuable candidates against obesity.

Lactic acid bacteria are also able to produce conjugated linoleic acids, gamma-aminobutyric acid (GABA) and may contribute to signal the neuroendocrine and vascular systems. More studies are necessary to determine the best strains, optimal dose, and treatment time to achieve beneficial outcomes for obesity, type-2 diabetes, non-alcoholic fatty liver disease (NAFLD) and decipher the corresponding mechanism(s). Collectively, those data should shed light on selected probiotic strains as important tools to prevent and treat patients with metabolic disorders and cardiovascular diseases.

### Antimicrobial Properties

Besides the widely known lactic acid and H_2_O_2_-mediated anti-bacterial, anti-viral, and anti-fungal properties of LAB, specific bacteriocin-based mechanisms can control bacterial growth in distinct environments. Bacteriocins are ribosomally synthesized peptides or proteins that exhibit bactericidal or bacteriostatic activities (Leroy and De Vuyst, 2004; Cotter et al., 2005). The classification of bacteriocins is a long-term matter of debate. Lately, a large scientific consortium proposed a new classification in which post-translationally modified bacteriocins of less than 10 kDa are considered as Ribosomally synthesized and Post-translationally modified Peptides (RiPPs) (Arnison et al., 2013). This new classification was slightly modified to embrace all bacteriocins leading to three classes, where class I are RiPPs, class II are unmodified bacteriocins (less than 10 kDa), and the class III are large bacteriocins (Alvarez-Sieiro et al., 2016).

Bacteriocins are found in almost every examined taxa. Their likely ubiquitous nature suggests they play a major role in shaping bacterial communities: they may serve as anticompetitors preventing invasion of a bacterium into an established community and could reciprocally allow a bacterium to invade a community (Riley and Wertz, 2002). Intuitively, their activity should lead to diversity reduction; however, experimental and modeling data suggest that under certain circumstances, bacteriocin production can promote diversity providing that taxonomic diversity is mirrored by bacteriocin-encoding gene diversity (Abrudan et al., 2012).

There are three major already established or future applications of bacteriocins. They can be used as biopreservative agents, as probiotic-promoting factors, and as antibiotics. Although two bacteriocins, nisin and pediocin, are allowed as food preservatives, bacteriocins are mainly indirectly used through producer strains. These strains are included into ready-to-eat food in order to produce bacteriocins *in situ* and are mainly used to target *Listeria monocytogenes* (Alvarez-Sieiro et al., 2016). Bacteriocin-producing LAB are also promising probiotic candidates for humans and animals (Dobson et al., 2012). Proof of concept was demonstrated by showing that bacteriocin production by *L. salivarius* UCC118 allows protecting mice from *L. monocytogenes* infection (Corr et al., 2007). Several studies revealed that bacteriocin production results in changes in the gut microbiota structures (Murphy et al., 2013; Umu et al., 2016) leading to the idea of using bacteriocins as tools for targeted manipulation of gut microbiota (Murphy et al., 2013).

Besides biopreservation and probiotic-promoting factors, bacteriocins are considered as a means to fight against emerging multidrug resistant pathogens (Cotter et al., 2013). A promising strategy consists in combining the use of bacteriocin with other antimicrobials to reduce the frequency of resistant variants appearing and/or to increase the antimicrobial potency (Mathur et al., 2017).

All these possible applications fuel active research, aiming at identifying new bacteriocins. Despite the tremendous literature describing new bacteriocins, new ones with interesting novel structures and unique activity are still being discovered. The challenge is now to design strategies that allow to avoid already reported bacteriocins. The traditional approach, isolating a strain exhibiting antagonistic activity followed by purification of the active compound prior characterization, is time-consuming, expensive, and tedious. Therefore, new workflows were developed that include steps dedicated to assess the novelty of bacteriocin candidates. One strategy combines liquid chromatography/mass spectrometry with principal component analyses of the antimicrobial spectrum of each bacteriocin-producing LAB strain (Perez et al., 2014). There are also *in silico* strategies based on genome mining that allow identifying candidate genes specifying putative bacteriocin proteins with novel structures (Collins et al., 2017).

### Detoxifying Properties of LAB

The LAB, among other bacteria, have been suggested as tools for detoxification of several xenobiotics and pollutants such as pesticides, toxins, and heavy metals. Whereas bioremediation is actively used in the environmental industry for many years, *in vivo* applications of LAB to reduce contaminant bioavailability are only in the early stages of development. Proof of concept has already been established, however, using distinct animal models and pilot clinical studies (Trinder et al., 2015; Wang Y.S. et al., 2016). For example, specific *Lactobacillus spp.* and *Leuconostoc* isolated from kimchi possessing an organophosphorus hydrolase gene were able to degrade pesticides such as chlorpyrifos and parathion (Islam et al., 2010). In this context, resistance to insecticides (chlorpyrifos, fipronil) was associated with the presence of *Lactobacillales* in the midgut of insects (Xia et al., 2013). Recent data support the use of an *L. rhamnosus* strain to reduce absorption and subsequent toxicity of organophosphate pesticide and neonicotinoid to *Drosophila melanogaster* (Trinder et al., 2016; Daisley et al., 2017, 2018). These are encouraging results that might be helpful in the fight against the current worldwide environmental threat of bees dying from these toxic compounds. They also suggest further examining the use of supplements for human, livestock, or apiary foods with selected probiotic microorganisms.

Lactic acid bacteria have also shown potential in mitigating toxic effects of distinct mycotoxins such as aflatoxins (B1, F1 and M1), patulin, ochratoxin A, and deoxinivalenol in food and feed (Fuchs et al., 2008; Ahlberg et al., 2015). Certain *Pediococcus* spp. have mycotoxin adsorbing and degrading properties (Martinez et al., 2017). Reducing aflatoxin bioavailability was reported in rats (Hernandez-Mendoza et al., 2011; Nikbakht Nasrabadi et al., 2013) and in mice (Jebali et al., 2015), but yet no clinical data are available on the use of LAB as detoxifying probiotics in humans. Other toxic substances can be handled by LAB. Acrylamide-binding ability by LAB was found to be both concentration- and strain-dependent, *in vitro* (Serrano-Nino et al., 2014) as well as in a gastric digestion stimulator (Rivas-Jimenez et al., 2016); the binding was mostly based on teichoic acid properties of specific strains. These results are promising for further actions and research to reduce exposure and bioavailability of such carcinogenic compounds.

Bioremediation of heavy metal (HM) and other hazardous and toxic metals such as chromium (CrVI) and aluminum, and, to a lesser extent, copper and cobalt, is challenging. The bioremediation is based on specific capacities of microorganisms to immobilize and/or inactivate pollutants by various passive (biosorption, complexation) or active transport (internalization, efflux/uptake ratio) mechanisms, including distinct bioprocesses (oxido-reduction, demethylation, e.g., for CH3-mercury) to lower HM bioavailability. Efforts have mostly focused on LAB and related species, and these have shown that selected lactobacilli (and bifidobacteria) can sequester lead (Pb) and cadmium (Cd) divalent cations and mercury (Hg) salts (Ibrahim et al., 2006; Halttunen et al., 2007, 2008; Bhakta et al., 2012). A key point is the huge variations in binding capacity among LAB species and even strains (Kinoshita et al., 2013). This variability is partly caused by the high diversity of ionisable compounds on the surface of Gram-positive bacteria (Lebeer et al., 2010). For example, EPS and teichoic acids comprise negatively charged groups (hydroxyl, carboxyl, phosphate) as potential ligands for divalent metal cations (such as Pb^2+^, Cd^2+^, Hg^2+^). In contrast, peptidoglycan and a mosaic of specific surface proteins (e.g., S-layer proteins) have positive charges, which can explain affinity toward arsenate As(V) (Schär-Zammaretti and Ubbink, 2003). Since the last decade, bioremediation of HMs and tolerance of humans to HMs through microbial processes using food-grade microorganisms have been highlighted (Monachese et al., 2012; Kumar et al., 2018). This concept can also be applied *in vivo* ‘inside the digestive tract.’ Commensal intestinal microorganisms play a positive role in the interactions with HMs, as demonstrated by we and others (Nakamura et al., 1977; Breton et al., 2013). Provided that microbial species meet criteria for safe dietary use, transitory microorganisms may do at least as well or even better than the resident flora (Plé et al., 2015). Various selected food microbes can thus prevent the absorption of HMs by the body and remove them upon defecation. Promising proof of concept of efficiency was demonstrated in preclinical models of acute and chronic HM toxicity in mice for cadmium (Zhai et al., 2013, 2014), chromium (Younan et al., 2016), and aluminum (Yu et al., 2017). Likewise, a first clinical pilot study showed reduced circulating levels of toxic HMs in pregnant women and children living in a contaminated area: by using a designed fermented milk with a selected probiotic *L. rhamnosus*-supplemented yogurt the bioaccumulation of mercury and arsenic could be reduced (Bisanz et al., 2014). This work clearly illustrates the promising concept of using probiotics in a nutritional strategy against xenobiotics.

### Other Attributes and Emerging Properties of LAB

Opportunities to use LAB for health are quite unlimited as the role of intestinal microbiota has now been clearly highlighted in various diseases and pathologies, including those affecting organs at distant sites, cancers, neurologic, and psychological disorders. Some examples are abdominal hyperalgesia and pain that can be controlled by specific strains of lactobacilli targeting nociceptive signals and these strains have been proposed in treatment of irritable bowel syndrome (Ringel-Kulka et al., 2014; Perez-Burgos et al., 2015). Interestingly, in the 2014-clinical study, an *L. acidophilus* strain was clinically effective, but failed to attenuate pain in patients when co-administered with another bacterial strain (*Bifidobacterium lactis*). The results suggest a diversion effect and caution should be taken when using mixtures of strains. In cancer, a higher enterococci count was correlated with a lower risk of colorectal cancer development. Some reports showed an inverse correlation of fecal enterococci with colon adenomas (Kawano et al., 2018), while another study suggested a deleterious role of *E. faecalis* in colorectal cancer development (Huycke et al., 2002). Selected LAB, such as *Enterococcus hirae*, can be also used to boost cancer chemotherapy (Daillère et al., 2016), while appropriate strains may also play a role in immune cell regulation and exhibit anti-oxidative and antigenotoxic effects.

Other examples of innovative use of LAB as probiotics are in modulating central nervous system functions (Wang H. et al., 2016) and behavior symptoms such as chronic fatigue, depression, and anxiety (Slykerman et al., 2017), although clinical data are yet not fully convincing (Pirbaglou et al., 2016; Ng et al., 2018). The results have led to the definition of ‘Psychobiotics’ as a novel class of psychotropic treatments employing bacteria with neuroendocrine and behavioral properties (Sarkar et al., 2016). Functional magnetic resonance imaging has been used for the first time to measure brain activation triggered by probiotic LAB (Bagga et al., 2018). A deeper understanding of the relationships between the LAB (within the gut or ingested) and the host, if they do really exist and what are the mechanisms, is required to develop microbial-based therapeutic strategies for brain disorders. Indeed, emerging applications are still on examination, partly due to the lack of consistent studies, appropriate study designs, and the selection of the proper strain(s).

## General Conclusion

**FIGURE 1:**
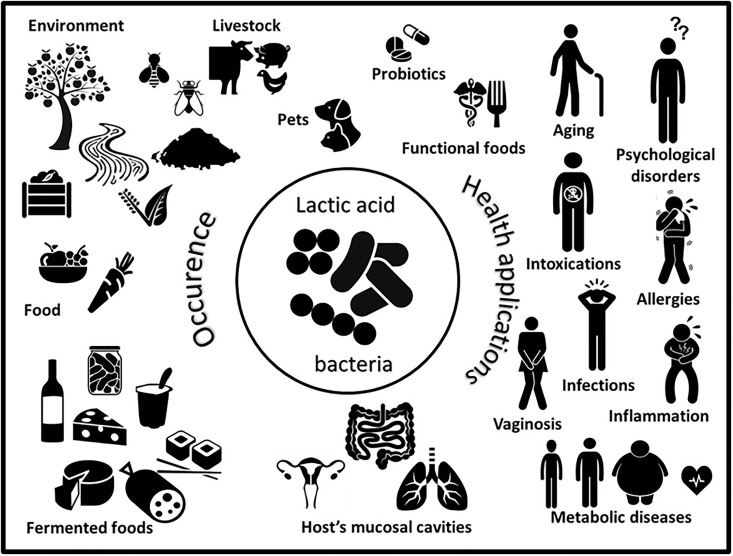
Schematic representation of the worldwide occurrence (left) and multiple health applications (right) of lactic acid bacteria.

The LAB are multifaceted microorganisms that have existed on earth for several millions of years, with tens of thousands of years of shared history with animals and humans. They have been used for the production of fermented foods for centuries, and more or less actively developed as probiotics for several decades (Figure 1). LAB may strongly be part of the health concept for livestock rearing and in food and feed production. An outstanding effort has been made these last years, using extended omics approaches, to build the knowledge and further tools to elucidate the contribution of LAB in health and diseases. We are now facing the daunting task of integrating of all this information for general application as well as for individual use (“my personalized LAB”). Indeed, separately, LAB can either contribute to induce Th1 or Th2 immune responses; they may also induce specific or non-specific regulatory T cells, which may or may not be required by the host. Similarly, LAB have the potential to favor either weight loss or weight gain. LAB abundance is sometimes diminished or increased depending on diseases. The central role of LAB within the microbiota, providing antimicrobials, also raises the question of the control of ecological niches, which can be advantageous or not (Berstad et al., 2016; Hegarty et al., 2016). Berstad argued that ‘we should stop thinking of LAB as always being friendly.’ Indeed, few data exist on the long-term impact of LAB, considering their possible capacity to destabilize the microbiota, and the “paradox” to use them empirically in multiple pathologies and combined (metabolic, immune, psychological) disorders. Nevertheless, LAB are our obligate partners and we have to cope with these microorganisms. Dissociating the common and specific interactions of LAB strains, -species and -genera within the whole of the microbiota in which they partake is still necessary to identify regulatory mechanisms, respectively, involved in distinct organs, systems, and hosts. Definitely, integrative system biology approaches are required to achieve the ultimate goal of applying LAB for personalized medicine. It comprises using omics technologies on the LAB as well as on the host and including foodomics and nutrigenomics (Kussmann and Van Bladeren, 2011; Bordoni and Capozzi, 2014), together with appropriate basic and integrative models and tools (Fritz et al., 2013; Daniel et al., 2015; Papadimitriou et al., 2015) to appraise the overall functionality of LAB.

## Author Contributions

BF, FG, MT, and FB prepared the manuscript and all co-authors contributed to editing and critical reviewing thereof.

## Conflict of Interest Statement

The authors declare that the research was conducted in the absence of any commercial or financial relationships that could be construed as a potential conflict of interest.
